# Supplementary material for: Motion direction is represented as a bimodal probability distribution in the human visual cortex
Source: Nat Commun. 2023 Nov 22;14:7634. doi: 10.1038/s41467-023-43251-w (PMC10665457; doi:10.1038/s41467-023-43251-w)
Supplement: Supplementary file 1 — Supplementary information [file 41467_2023_43251_MOESM1_ESM.pdf]

Motion direction is represented as a bimodal probability distribution in the human visual cortex

Andrey Chetverikov Janneke F.M. Jehee

Supplementary figures

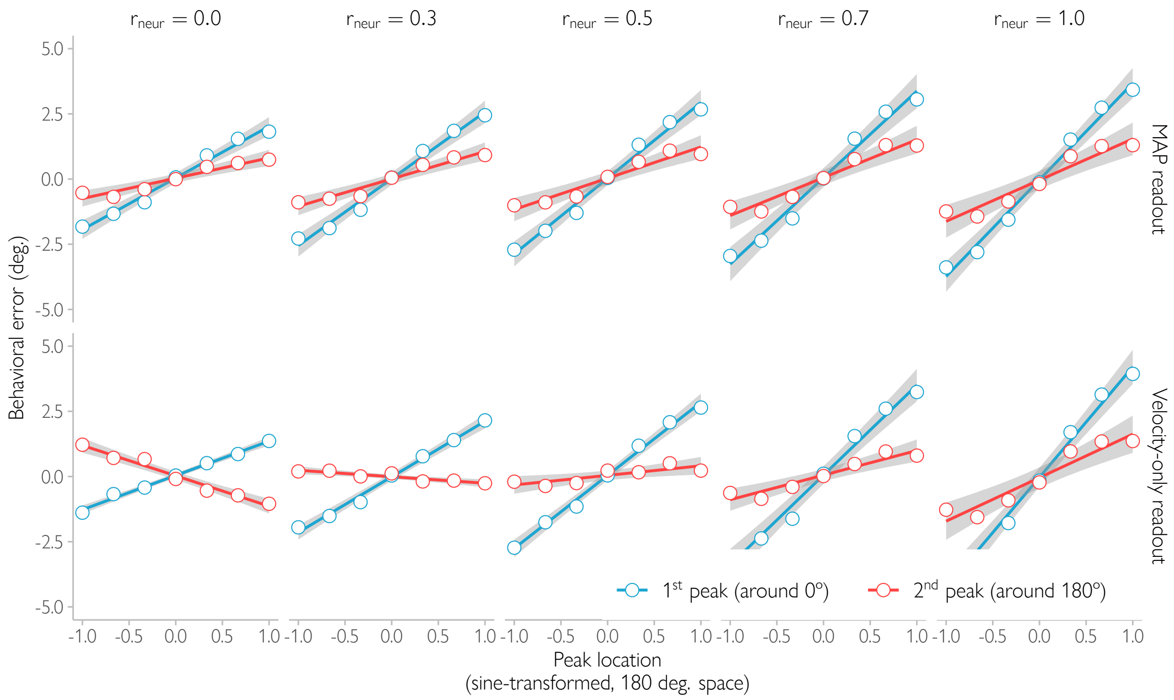

Supplementary Figure 1. **Non-independent neural noise**. The panels show the relationship between the behavioral response and the peak locations for a model assuming that the neural signals for motion and orientation are not independent. Different levels of correlation *r* between the two signals are shown. For the Bayesian observer model that uses both velocity and spatial orientation signals to infer motion direction ('MAP readout'), the location of either peak in the decoded posterior is positively correlated with the direction and magnitude of the behavioral errors, regardless of correlation strength. In contrast, for an observer who has a bimodal probabilistic representation of motion direction but uses only velocity (and not spatial orientation) signals ('velocity-only readout'), the correlation between the second peak location and behavioral errors is negative for weak correlation, absent for moderate correlation, and is positive only in case of strong correlation. Note that the 'velocity-only readout' model is inconsistent with the results of the follow-up behavioral study. Note that, as expected, the first column in this figure matches the results in Fig. 2c, because the correlation parameter *r* equals 0 here, so the measurements are independent. Please see Supplementary methods, section 3, for further detail on the simulations. In all panels, circles show the mean simulated error, and the lines show the fit of the linear regression model with shaded regions showing 95% confidence intervals.

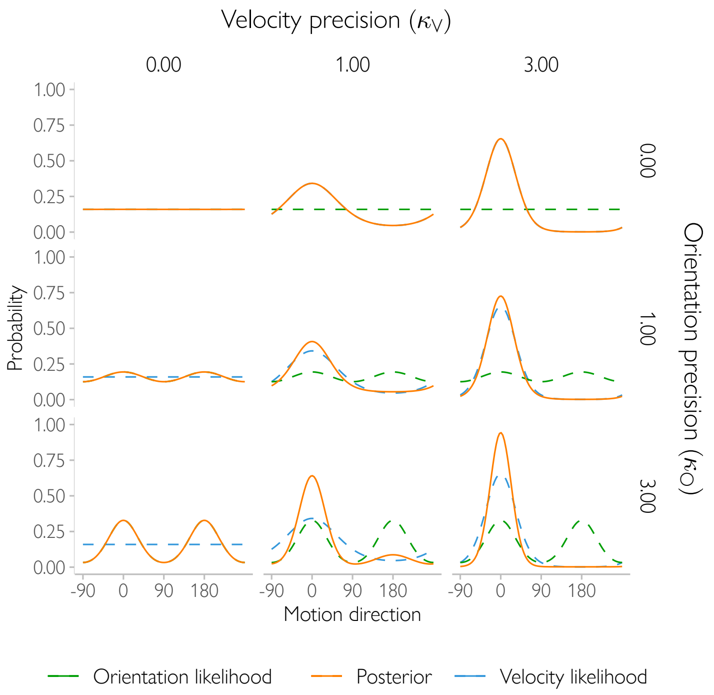

Supplementary Figure 2. **The shape of the posterior distribution as a function of the variance in the velocity and orientation measurements for the Bayesian observer model**. Shown is the likelihood given the velocity measurement (blue, dashed line), and the likelihood given the spatial orientation measurements (green, dashed line) for different levels of variance (precision parameter κ) in the two measurements. Because these two likelihoods are multiplied to compute the posterior distribution (orange, solid line), the shape of the posterior depends on the shape of the two likelihoods. When the variance of the velocity measurements is relatively low (κ_V is high, right column), resulting in a narrow likelihood, the posterior becomes a unimodal von Mises distribution. In contrast, if the variance of the velocity measurements is high (κ_V is close to zero) and the variance of the orientation measurements is low (κ_O is high), the posterior becomes bimodal with two identical peaks at the true and the opposite directions of motion (lower-left plot). Finally, when both the velocity and orientation measurements are highly variable, resulting in very broad likelihoods, the posterior distribution becomes close to uniform.

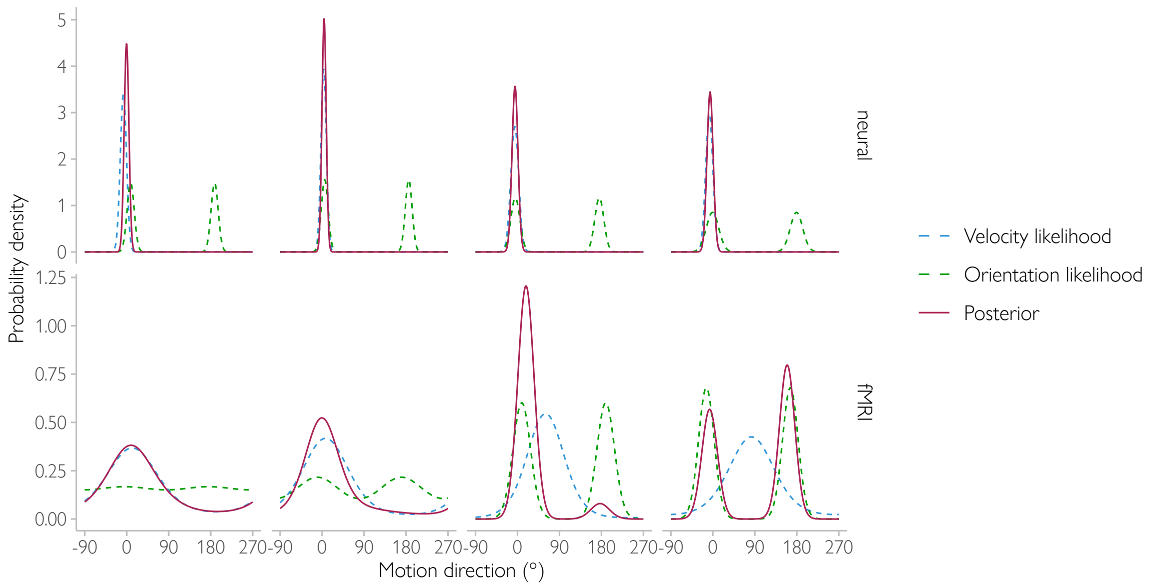

Supplementary Figure 3. **Concurrent BOLD bimodality and neural unimodality**. Bimodality in the posterior distribution decoded from fMRI data can arise even if the neural posterior distribution that is read out for behavior is unimodal. This happens because of the non-neural sources of noise that are included in the fMRI signal. Here, the top row shows the simulated 'neural' likelihood and the corresponding posterior (magenta) computed from these likelihoods (blue: velocity, and green: orientation) for four example trials (each column shows one trial). Because there is comparatively little noise at the neural level (as we assume is the case for the fMRI experiment presented in the paper), the neural posterior that is read out for behavior is unimodal. However, measuring cortical activity with fMRI introduces additional noise. This additional noise is combined with the neural measurements (Eq. 30), and results in wider likelihoods, which in turn, correspond to wider and sometimes bimodal (third and fourth columns) posterior distributions measured with fMRI (bottom row).

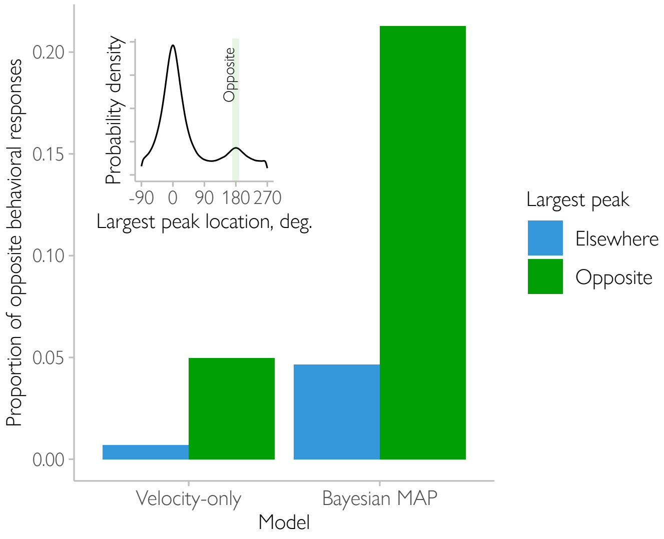

Supplementary Figure 4. **The predicted relationship between the decoded posterior distribution and the observer's behavioral responses for low coherence stimuli**. To assess whether it would be helpful to collect additional fMRI data for low coherence stimuli, we simulated decoded posterior distributions and behavioral responses for this scenario. We were specifically interested in a potential relationship between the location of the decoded posterior's largest peak and opposite behavioral responses when motion coherence is low and the neural posterior is bimodal. Accordingly, for both the velocity-only model and the Bayesian model, we divided the simulated trials over two bins: one for which the largest peak of the decoded posterior fell 'opposite' to the presented direction of motion (i.e., 170-190 degrees away from the presented direction of motion, green), and another bin (blue) for all the other trials. The inset shows the across-trial distribution of the largest peak location, with the green region depicting the 'opposite' bin. Within each bin, we then calculated the fraction of trials on which the model observer gave the 'opposite' behavioral response. The bar graphs show the obtained results. While the Bayesian model predicts a larger overall fraction of opposite behavioral responses than the velocity-only model, both models predict that the fraction of opposite behavioral responses is much larger when the larger peak of the decoded posterior distribution is located close to the opposite direction of motion than when it is located elsewhere. Interestingly, the increase in the fraction of opposite behavioral responses (compare blue with green) is even larger for the velocity-only than the Bayesian observer. Because both models predict a link with behavior, we conclude that analyzing fMRI data for low-coherence stimuli would not enable us to further adjudicate between the two models. Please note that merely analyzing the shape of the posterior in cortex for low coherence stimuli (i.e., without linking this to behavior), would similarly not allow for further adjudication between the two models. The details related to these simulations are provided in Supplementary Methods, section 3. This section also provides an intuition for why we observe these results.

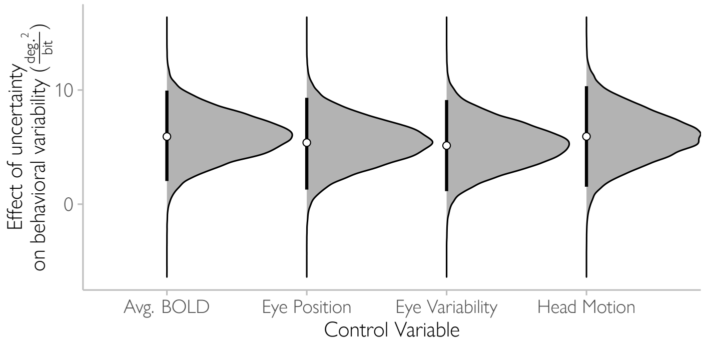

Supplementary Figure 5. **Control analyses**. The effect of trial-by-trial uncertainty on behavioral variability was estimated after controlling for mean BOLD amplitude in the ROI, gaze fixation position, variability of gaze fixation positions, and head motion. The hierarchical regression model was identical to the main analysis (where decoded uncertainty and the absolute distance to the nearest cardinal direction were included as independent variables), except that the corresponding control variable was included as an additional predictor both as a fixed and as a random effect. We aimed to assess whether the addition of the control variable would eliminate the effect of uncertainty on behavioral variability. This was not the case: decoded uncertainty reliably predicts behavioral variability, regardless of the control variable included. This rules out simple explanations in terms of the amount of attentional effort put in the task (as mean BOLD amplitude tends to increase with attentional effort, see, e.g., [1]) or the amount of eye movements. Circles indicate the estimated effect (i.e., the regression coefficient) with bars showing 95% HPD credible intervals (none of the intervals includes zero). The associated distribution of the samples from the regression model posterior is shown in gray.

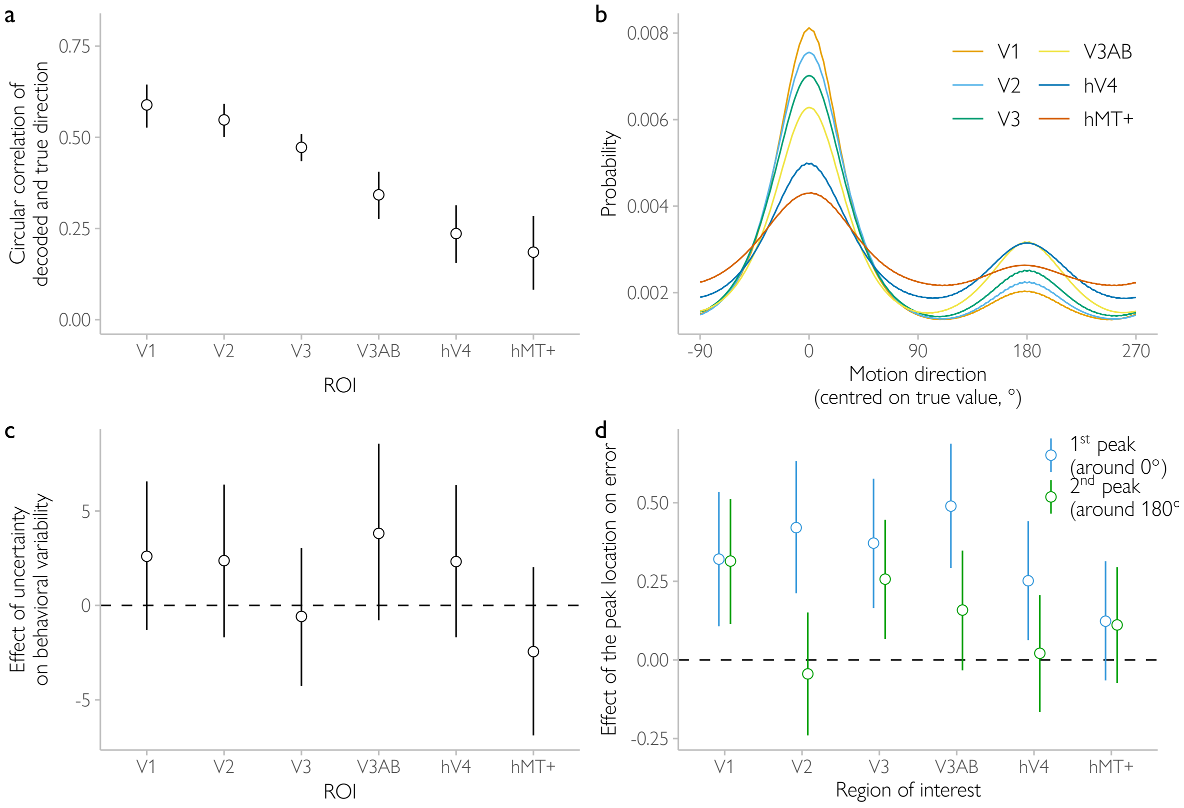

Supplementary Figure 6. **Results separated by ROI**. **a** Circular correlation between the decoded and presented direction is above chance levels for all ROIs (all *BF* > 340). Circles show the mean and bars show 95% confidence intervals. **b** Posterior distributions decoded from individual ROIs (averaged across trials and all subjects) show the predicted bimodal pattern with peaks at the presented and opposite direction of motion. For all ROIs, the posterior distribution was bimodal with peaks close to 0 and 180° for the majority of observers (bimodal for 12 observers in V1, 15 observers in V2 and hV4, 16 in V3 and hMT+, and 18 observers in V3AB). **c** The effect of uncertainty (*b*, the estimated regression coefficient) on behavioral variability in trial-by-trial analyses estimated with a hierarchical Bayesian regression (not significant for any of the ROI taken individually) following the same procedure as described for the analyses of all ROI combined (cf. Figure 3c, which shows the significant effect for all ROIs combined). **d** The effect of the peak locations (*b*, the estimated regression coefficient) on behavioral errors in trial-by-trial analyses estimated with a hierarchical Bayesian regression following the same procedure as described for the analyses of all ROI combined (cf. Figure 4c). The peak closer to 0° (corresponding to the larger peak in the averaged posterior distribution in b) is correlated with the direction and magnitude of behavioral errors in all ROIs (all *BF* ≥ 10.62) except for hMT+. The peak closer to 180° (corresponding to the smaller peak in the averaged posterior distribution in b) is reliably correlated with errors only in V1 and V3 (*BF* = 239.19 and *BF* = 51.94, respectively). Circles and bars in **c** and **d** show the estimated regression coefficient and 95% highest posterior density (HPD) credible intervals, respectively.

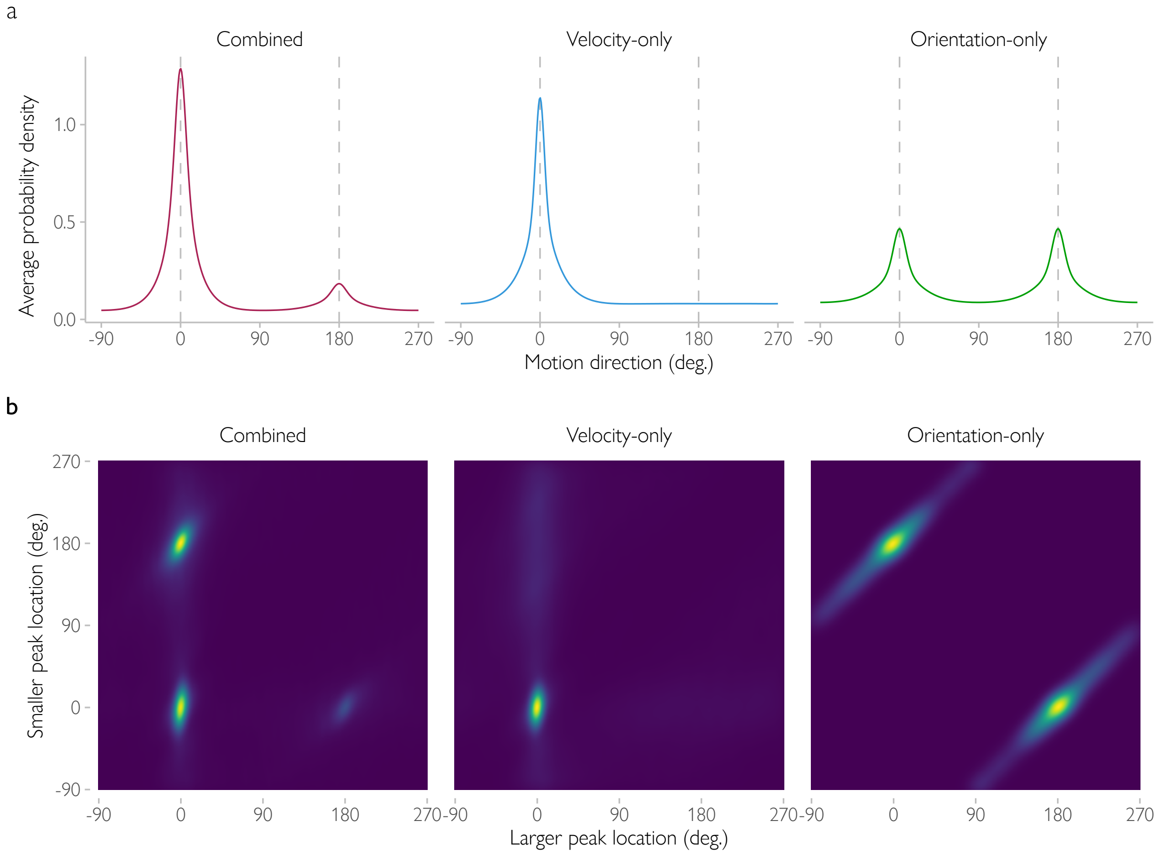

Supplementary Figure 7. **Simulated results for the cortical representation of motion direction**. **a** Decoded posterior distributions, averaged across trials. When decoding from a mixture of velocity- and orientation-tuned signals ('Combined'), the resulting posterior distribution is bimodal of shape, with a larger peak at the presented and a smaller peak at the opposite motion direction. In contrast, when information is extracted from velocity signals only ('Velocity-only'), the posterior distribution is unimodal. Using spatial orientation signals alone ('Orientation-only') produces a bimodal posterior with two equal-sized peaks. **b** To plot the joint probability distribution of peak location (shown), the locations of the two peaks in the posterior (estimated using a mixture of von Mises model as described in the Methods) were tabulated for each trial, and the joint histogram of possible location pairs was obtained across trials for each posterior type. Three clusters of trials can be observed for the joint probability distribution of the two peak locations for the Combined condition, in which brighter colors correspond to higher probabilities of observing a given combination of peak locations on a given trial. For a considerable portion of trials, the larger peak of the posterior distribution is located around the presented direction of motion, while the smaller peak is located around the opposite direction (i.e., a bimodal decoded posterior). In contrast, for the Velocity-only condition only a single cluster is observed with both the larger and the smaller peak location located around the presented direction of motion. Finally, for the Orientation-only condition, two clusters are observed with the larger and the smaller peak separated by 180 degrees. Please see Supplementary Methods, section 1, for further detail on the simulations.

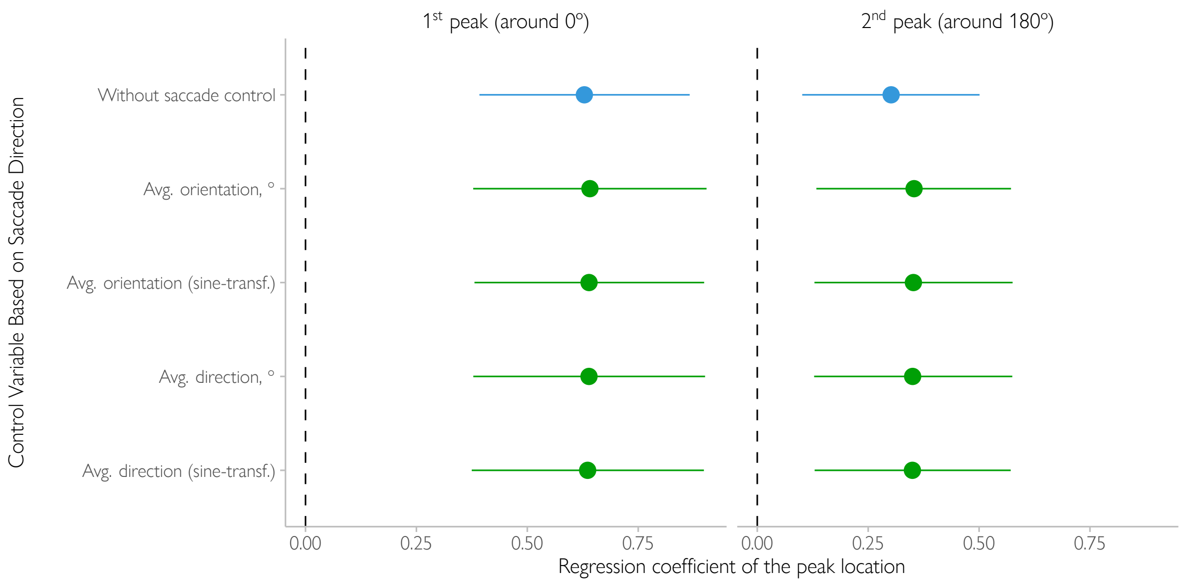

Supplementary Figure 8. **Control analyses involving saccade direction**. To ensure that the relationship between posterior peak location and the direction and magnitude of behavioral errors (Fig. 4c) is not due to the presence of eye-movements, we ran additional control analyses. Specifically, we included additional variables in our analysis to see if the relationship between the peak locations and the behavioral errors can be explained by these variables. The variables were computed over a narrow time window from 0 to 4.5 seconds after the start of the trial (note this corresponds to the same interval that we used for decoding, after accounting for hemodynamic lag). We used the mean saccade direction without any transformations ('avg. direction') and the mean sine-transformed saccade direction to probe for a potential circular relationship ('avg. direction (sine-transf.)'). Additionally, to test for a potential effect of back-and-forth saccades (i.e., if the dots are moving to the right, the observers might saccade to the right and then to the left, back to the fixation point), we included the average orientation of saccades ('avg. orientation') as well as the linearized version of saccade orientation ('avg. orientation (sine-transf.)'). For all control variables, the regression coefficient of peak location was reliably above zero and virtually the same as without the addition of the control variable (blue, top), indicating that saccade direction does not play a significant role in the observed relationship between the peaks of the decoded posterior and the observer's behavioral errors. Dots denote the regression coefficient, and bars show 95% highest posterior density (HPD) credible intervals.

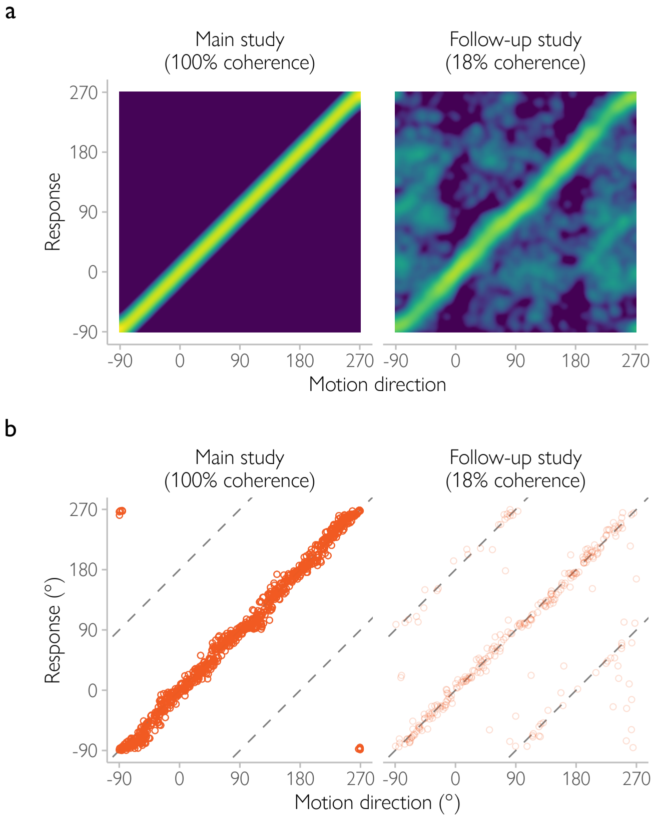

Supplementary Figure 9. **Behavioral performance in the main fMRI study and the follow-up behavioral experiment**. **a** Joint probability distribution of the presented motion direction and the observer's judgment, aggregated across observers. **b** Estimates of motion direction for an example observer (each dot represents a single trial). Note that for the follow-up study, the plots match Figure 4 (reproduced here for ease of comparison).

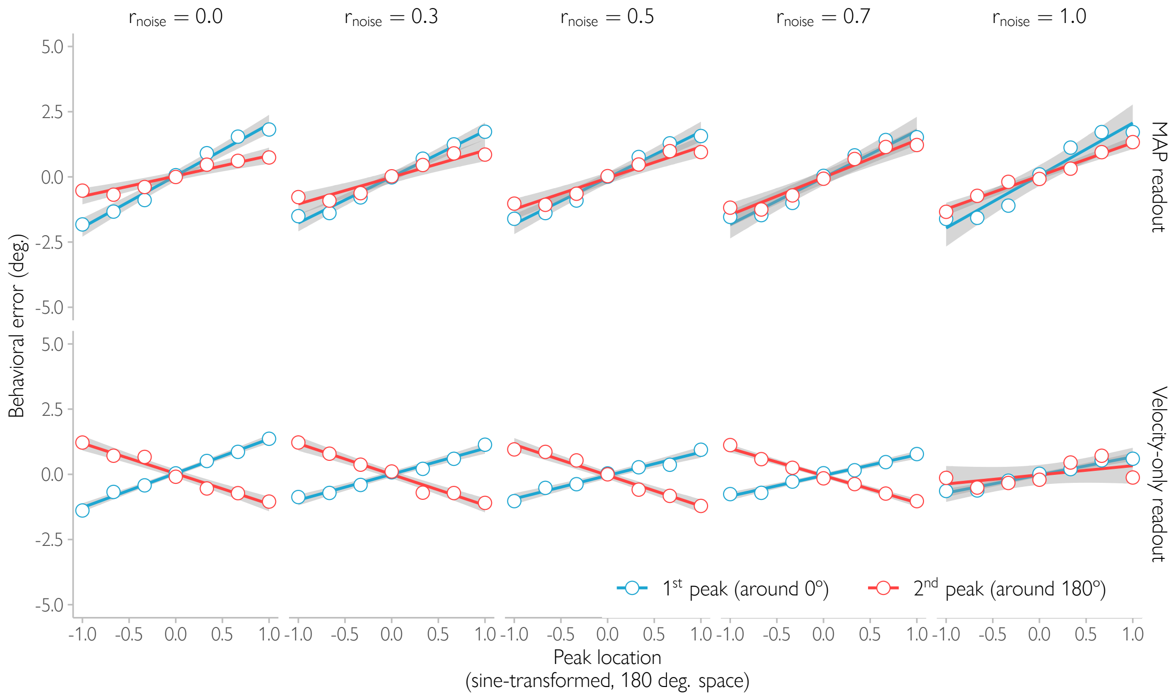

Supplementary Figure 10. **Neuroimaging noise that is non-independent**. The panels show the relationship between the behavioral response and the peak locations for a model assuming that the BOLD signal for motion and orientation is not independent due to non-neural sources of noise, such as fMRI scanner noise and physiological sources of noise, that affect both signals. Different levels of correlation *r* between the signals are shown. Please see the Supplementary methods, section 3, for detail on the simulations. For the Bayesian observer model that uses both velocity and spatial orientation signals to infer motion direction ('MAP readout'), the location of either peak in the decoded posterior is positively correlated with the direction and magnitude of the behavioral errors, regardless of correlation strength. In contrast, for an observer who has a bimodal probabilistic representation of motion direction but uses only velocity (and not spatial orientation) signals ('velocity-only readout'), the relationship between the second peak location and behavioral errors is negative, except for extremely strong correlation levels *r* that seem unrealistic for fMRI voxels. The circles show the mean simulated error, and the lines show the fit of the linear regression model with shaded regions showing 95% confidence intervals.

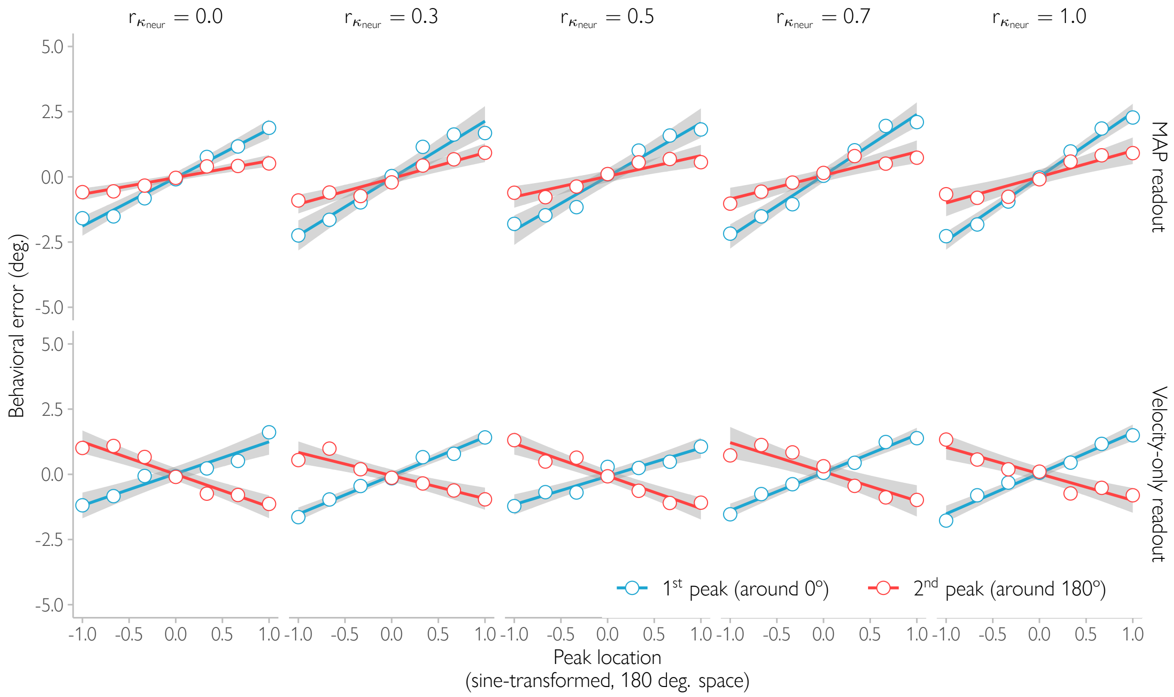

Supplementary Figure 11. **The variance parameters of the neural noise distributions are correlated**. The degree of uncertainty in the observer's motion and orientation estimates can covary due to, for example, fluctuations in attention. Here, we ask whether such covariation in uncertainty magnitude can explain the observed correlation between the peak locations and behavioral errors. Different amounts of correlation *r* between the motion and orientation uncertainty values are shown. Please see Supplementary methods, section 3, for further detail on the simulations. For the Bayesian observer model that uses both velocity and spatial orientation signals to infer motion direction ('MAP readout'), the location of either peak in the decoded posterior is positively correlated with the direction and magnitude of the behavioral errors, regardless of correlation strength. In contrast, for an observer who has a bimodal probabilistic representation of motion direction but uses only velocity (and not spatial orientation) signals ('velocity-only readout'), the correlation between the second peak location and behavioral errors is negative. The simulation results show that the velocity-only readout scenario is inconsistent with the empirical data. The circles show the mean simulated error, and the lines show the fit of the linear regression model with shaded regions showing 95% confidence intervals.

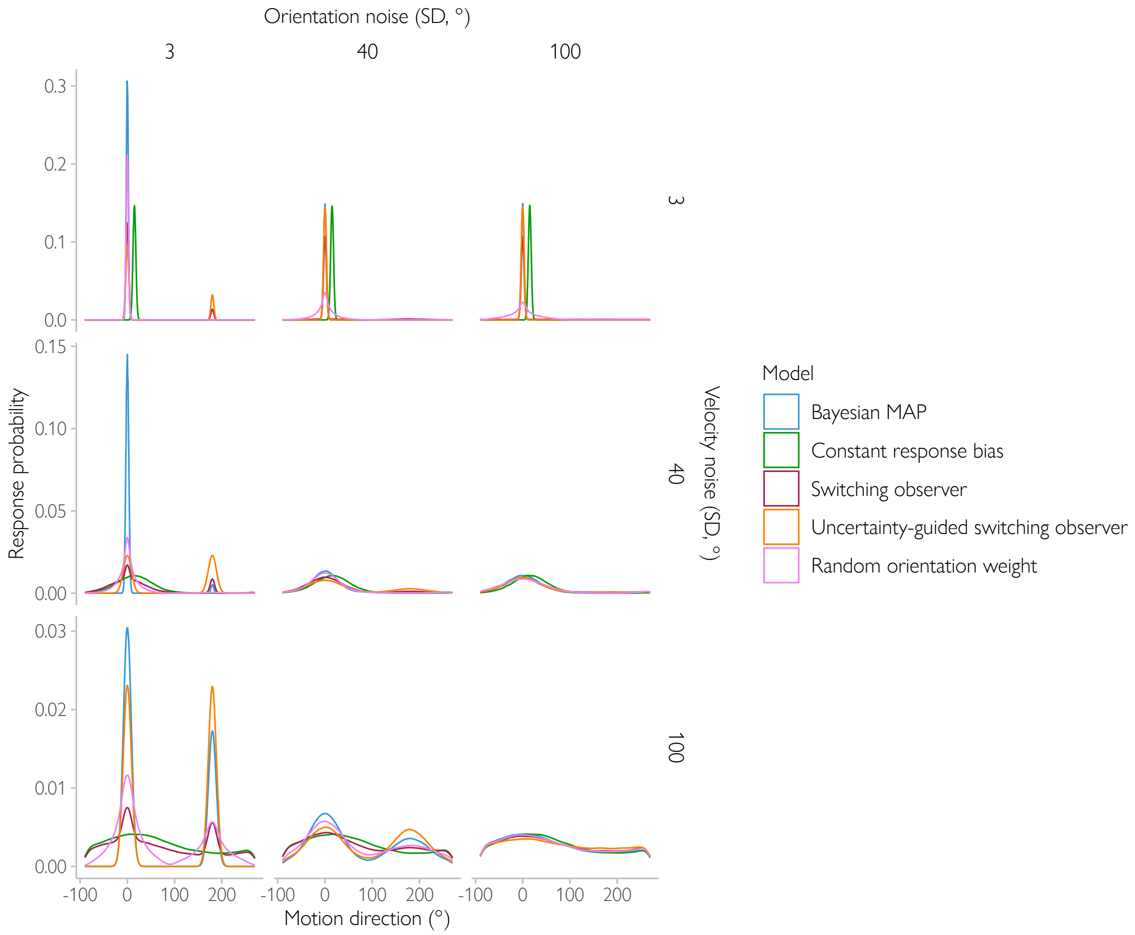

Supplementary Figure 12. **Simulated predictions for behavioral response distributions of alternative observer models**. Shown are the predicted behavioral response distributions for different amounts of sensory noise in the orientation and velocity measurements. The 'Bayesian MAP' model (blue, discussed in the paper) is shown for comparison. The 'constant response bias' model (green) assumes that behavioral responses are based on the velocity-only estimate alone with an arbitrary constant shift (here, +15°). Accordingly, the response distribution is shifted relative to the true stimulus direction at 0°, but never becomes bimodal. The 'random orientation weight' model (pink) combines spatial orientation and velocity signals while ignoring uncertainty; its response is a weighted average of the velocity-based estimate and the closest orientation-based estimate with randomly-assigned weights. This produces a bimodal response distribution when noise in the orientation signals is high. However, this model also predicts a very wide behavioral response distribution when uncertainty is low for velocity signals and high for orientation signals (upper-right corner). This is inconsistent with previous studies showing that observers perform well at slow motion speeds when orientation information is presumably very noisy or even absent[2]. A 'switching observer' (red) randomly switches between the velocity and orientation likelihoods on a proportion of trials (here, 20%). It exhibits bimodality in its response distribution, but the share of opposite behavioral responses is the same regardless of sensory noise. This is inconsistent with our results demonstrating opposite responses for low coherence (follow-up experiment) but not for high coherence stimuli (main experiment). The 'uncertainty-guided switching observer' (orange) selects the orientation or the velocity-based estimate based on their relative uncertainty[3]. This observer exhibits a higher probability of opposite responses than what is observed in our main experiment (high coherence stimuli), except when the orientation likelihood is flat (upper right plot). This is inconsistent with our fMRI results (Figure 4) which indicate that orientation signals are not only represented in cortex but also used by the participants in their behavioral estimates. Moreover, an already flat orientation likelihood cannot lead to the bimodal behavioral response distribution with decreased coherence observed in the follow-up experiment, making also this model inconsistent with our data.

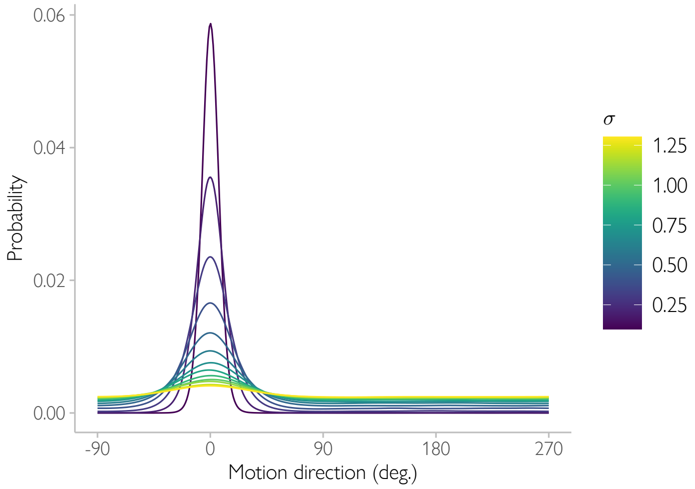

Supplementary Figure 13. **Posterior distribution of motion direction decoded from simulated responses of bimodally-tuned direction-selective neural populations**. Neurophysiological studies show that the tuning curves of direction selective neurons in V1 often have a smaller second peak around the direction of motion opposite to the preferred direction[4,5]. Here, we show that it is unlikely that these neurons alone could give rise to a bimodal posterior distribution at the level of voxels (the conclusions are similar for the behavioral response distribution). Please see Supplementary Methods, section 3, for detail on the simulations. As is evident from the figure, the posterior distributions decoded from the voxel responses (shown) was always unimodal and never bimodal, regardless of the level of tuning-dependent noise (σ^2, illustrated by different colors). Similar results were found for voxel-specific noise (τ_i^2). This further strengthens the hypothesis that the empirically observed posterior reflects the responses of cells whose spatial orientation receptive field runs parallel to the presented motion direction.

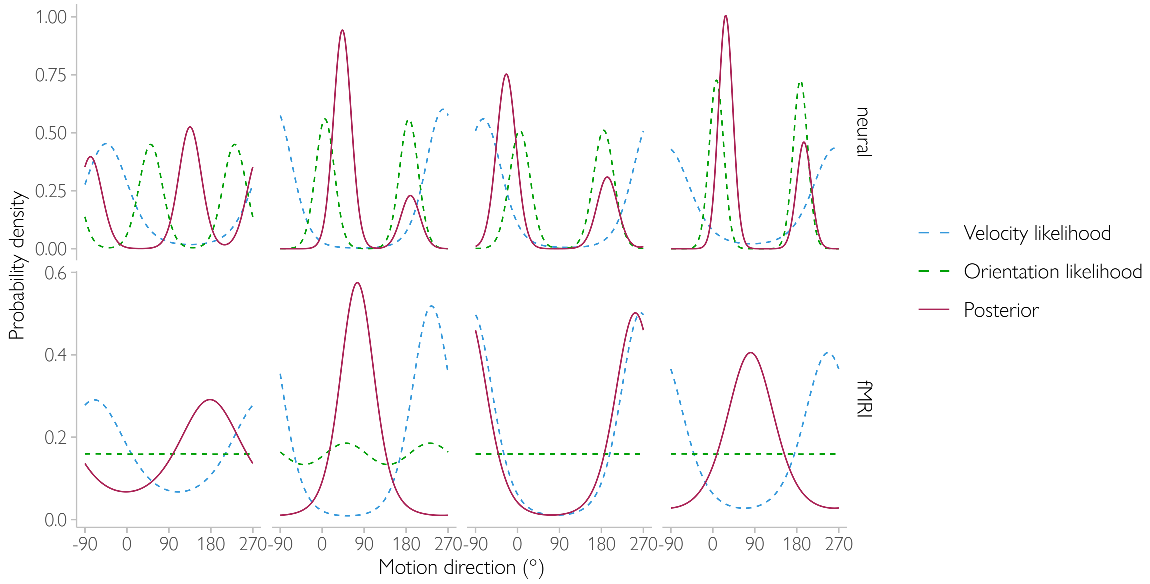

Supplementary Figure 14. **A unimodal decoded posterior does not necessarily imply a unimodal neural posterior**. Because the BOLD signal contains non-neural sources of noise, the fMRI posterior can be bimodal when the neural posterior that is read out for behavior is unimodal (Supplementary Fig. 3). Importantly, this also holds the other way around: the fMRI posterior can become unimodal when the posterior at the neural level is, in fact, bimodal. As shown here for four example trials (each column shows one trial), at higher noise levels, the posterior (magenta) computed from the neural signals becomes bimodal (top panels). However, the addition of non-neural sources of noise shifts the likelihoods for velocity (blue) and spatial orientation (green) and makes them wider. As a result, the fMRI posterior that is decoded from all of the signals combined can be unimodal (lower panels) even though the neural posterior is bimodal.

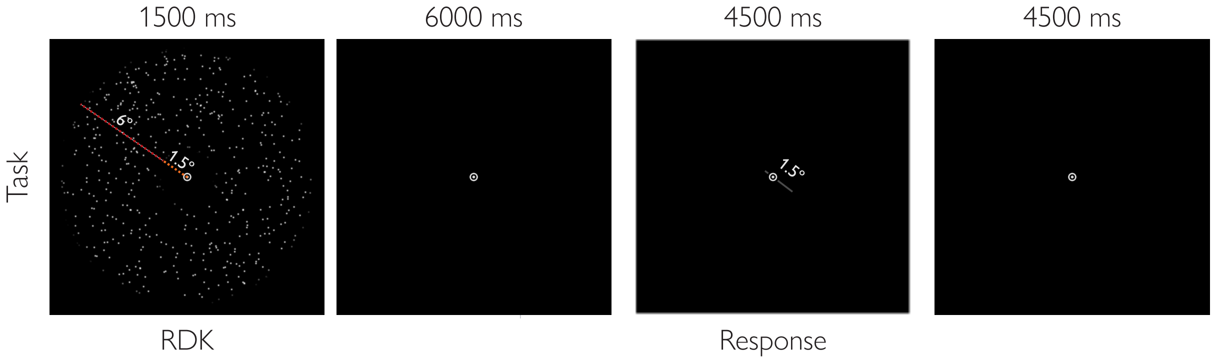

Supplementary Figure 15. **Events sequence within a trial**. A stimulus is presented for 1.5 s within an aperture (7.5 dva total radius) with an inner window (1.5 dva radius; the dashed lines were not shown to the observer), followed by a 6 s fixation interval. The observer then responds by rotating the bar on the screen (1.5 dva length) within a 4.5 s response window. After another fixation interval of 4.5 s, the next trial starts.

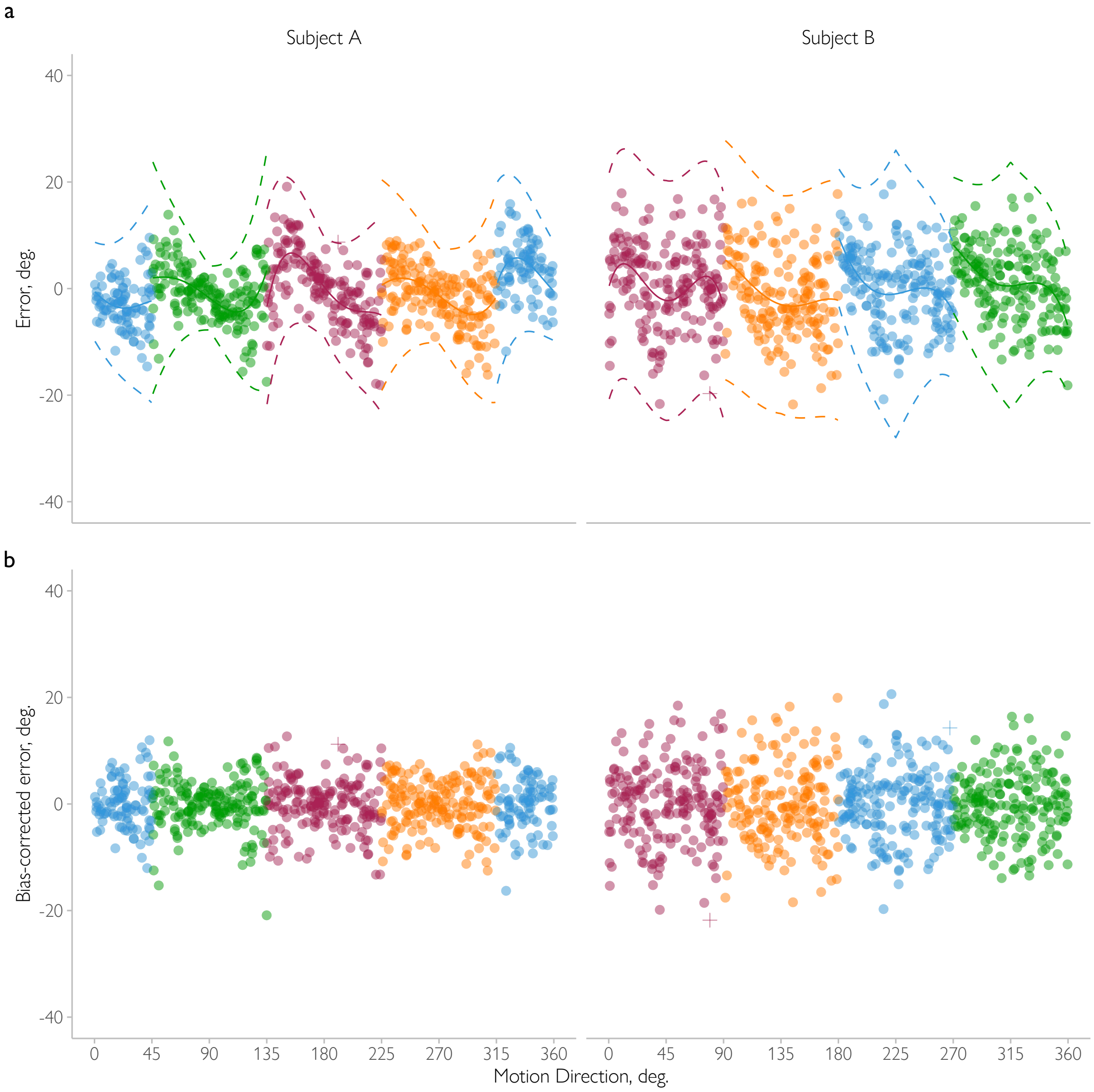

Supplementary Figure 16. **Removal of direction-dependent behavioral biases in two example observers**. **a** The behavioral responses (dots) of two example participants A and B. The behavioral data showed direction-dependent biases, which were removed prior to the main analyses, as follows. For each individual observer, we first determined the direction of their behavioral bias by fitting two models, which described either attraction or repulsion from cardinal directions. For the model that describes an attraction bias, the behavioral errors are expected to be close to zero at the cardinal directions. Accordingly, trials were split into four 90-degree bins (indicated by colors) centered at cardinal ({0, 90, 180, 270} degrees). For the repulsion biases, on the other hand, the errors are expected to be close to zero at oblique directions. Hence, for this model, the trials were divided into four 90-degree bins centered at oblique ({45, 135, 225, 315} degrees) directions. For each bin separately, and for each direction of motion within the bin, bias magnitude was determined by fitting a 4th degree polynomial with motion direction (computed relative to the bin center) as the independent variable and behavioral error as the dependent variable. To account for the heterogeneity of responses across motion direction (e.g., the oblique effect), the standard deviation of the behavioral errors was allowed to vary with distance to the polynomial's center (illustrated by dashed lines here, showing mean +/- 3 SD boundary). The best model (solid lines) of bias direction (as determined by their likelihood) was selected and subsequently used to remove the bias. All subsequent analyses were performed on the residuals of its fit (i.e., the difference between the predicted mean response for a given stimulus and the actual response). For subject A, the model with polynomials centered at oblique directions provides the better fit, while for subject B, the model with polynomials centered at cardinals is the better one. Errors that were larger than +/-3 times the predicted SD (shown with crosses) were considered outliers and not included in subsequent analyses. **b** The bias-corrected errors that were fed into subsequent analyses.

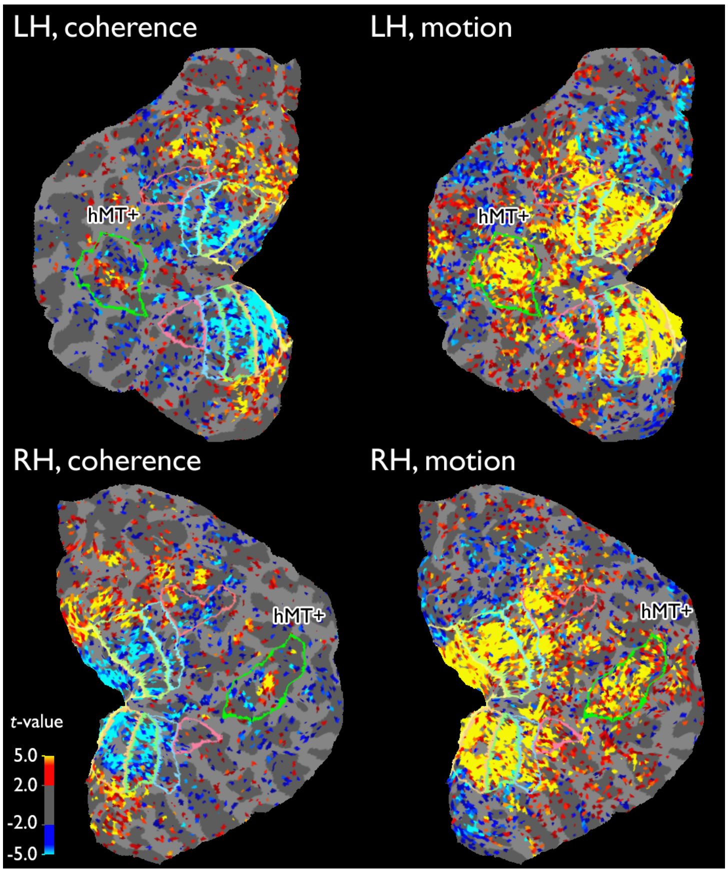

Supplementary Figure 17. **hMT+ localizer maps for an example participant** (LH: left hemisphere; RH: right hemisphere). hMT+ location (bright green) was determined based on a comparison of responses to coherent and random motion stimuli ('coherence') and a comparison between stimuli composed of moving or static dots ('motion'). See Methods for further detail. Colors indicate t-values for a given localizer contrast (threshold set here at 2.0 for consistency between subplots; hMT+ location was delineated with $p < .05$ threshold, FDR corrected). Other boundaries shown are those of V1(yellow), V2 (light green), V3 (blue) and hV4 (pink).

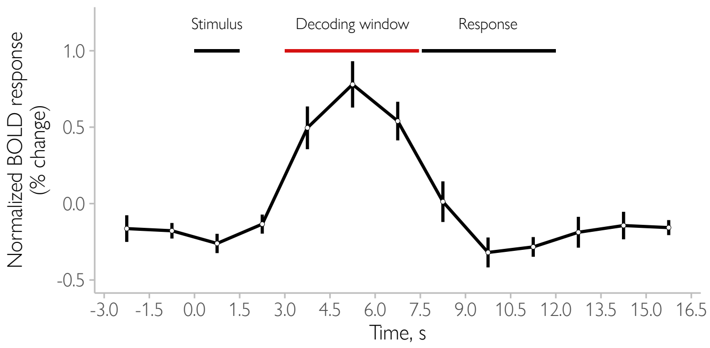

Supplementary Figure 18. **Time course of mean BOLD activity over the course of a trial in all ROIs combined**. Time points between 3 and 7.5 s were averaged and used in the decoding analysis. This relatively short window was chosen to ensure that no activity from the response window was included in the analyses. The decoding window aligns with the peak of the hemodynamic response function. Circles denote the mean, and bars show 95% confidence intervals.

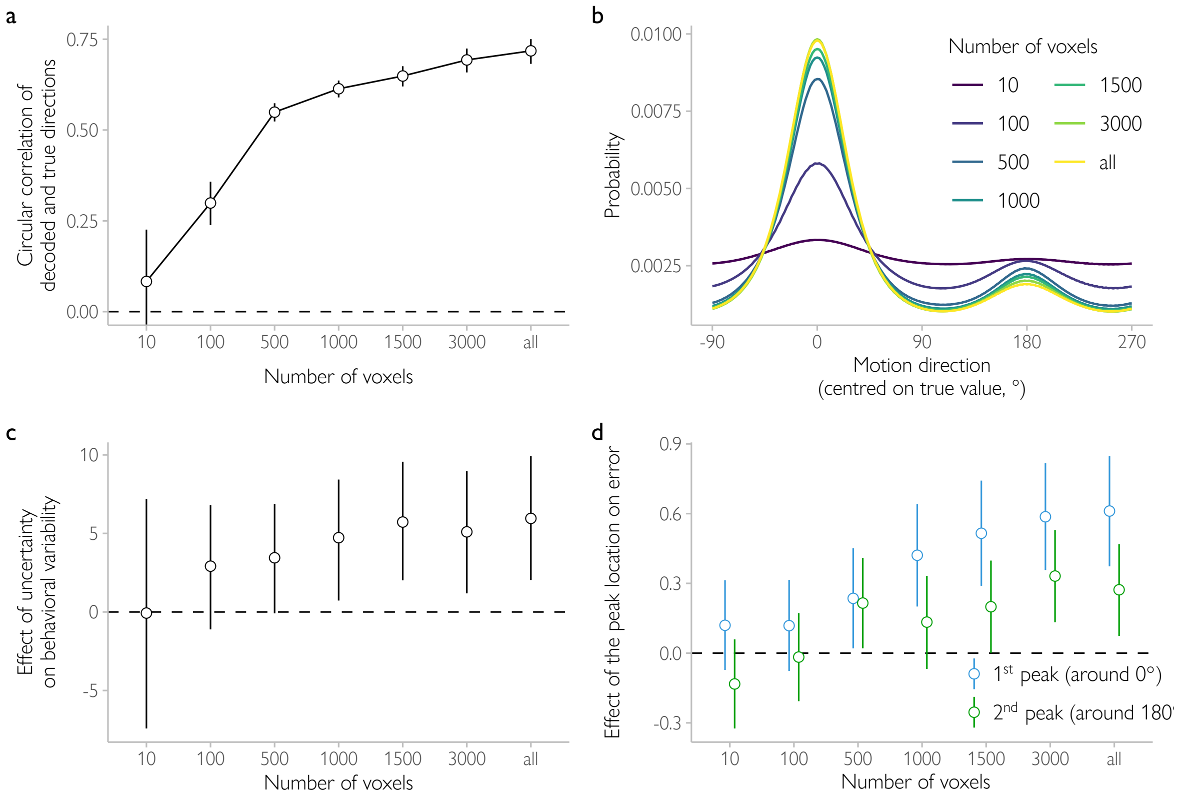

Supplementary Figure 19. **Control analyses using different numbers of voxels in the analyses**. Voxels were selected based on their response to the visual localizer stimulus, with the most active voxels selected first. **a** Decoding accuracy improved with increasing numbers of voxels, and remained relatively stable with 1000+ voxels. Circles show the mean correlation and bars show 95% confidence intervals. **b** The bimodality in the averaged decoded posterior distribution is observed with 100+ voxels. **c** The correlation between decoded uncertainty and behavioral variability in trial-by-trial analyses (after accounting for the effect of stimulus orientation) is positive for 500+ voxels ($BF \geq 3.01$). **d** The correlation between either peak location and behavioral error is observed for 500+ voxels ($BF \geq 8.90$ for the peak closer to 0°; $BF \geq 5.03$ for the peak closer to 180°, with the exception of $N = 1000$ voxels where $BF = 1.65$). Circles and bars in c and d show the estimated effect (i.e., the regression coefficient) and 95% highest posterior density (HPD) credible intervals, respectively.

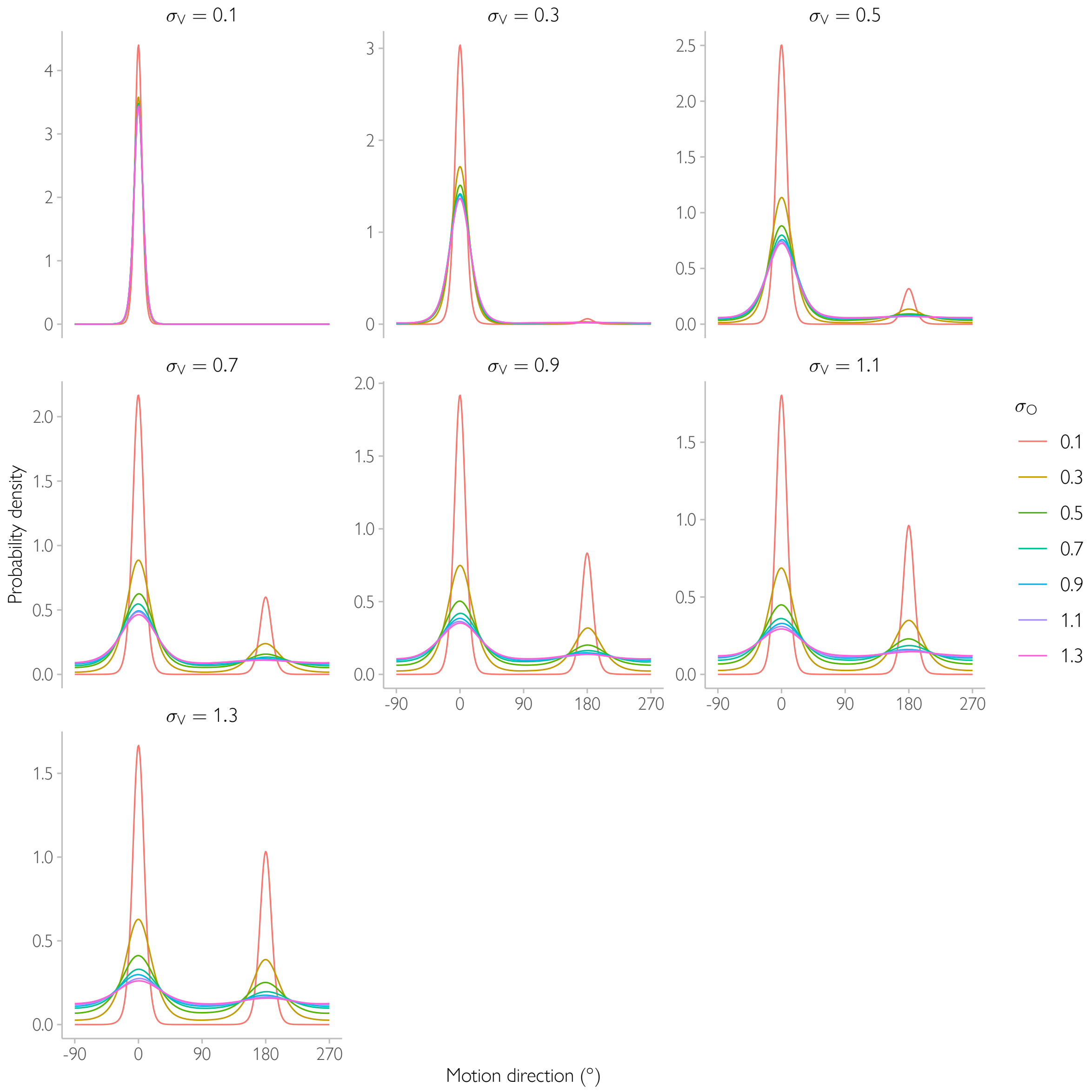

Supplementary Figure 20. **The shape of the posterior distribution decoded from simulated BOLD signals depends on uncertainty in the underlying orientation and velocity signals**. Using the generative BOLD model (Supplementary Methods, section 1), we simulated BOLD responses (Supplementary Eq. 16) for varying levels of noise (or uncertainty) in neuronal populations tuned to orientation (σ_O, panels) and velocity (σ_V, colors). As with the Bayesian observer model (Supplementary Fig. 2), the posterior (shown) becomes more bimodal when the uncertainty of velocity signals increases and the precision of orientation signals increases (uncertainty decreases). A separate set of simulations demonstrated that tuning-independent noise (τ) does not have a significant effect on the shape of the decoded posterior. Please see Supplementary Methods, section 1, for further detail on the simulations.

Supplementary Methods

1. Generative model of population-level response

To derive predictions for the cortical representation of motion direction, we additionally simulated population-level responses for velocity and spatial orientation and applied the decoding procedure as described in Decoding Analysis to estimate the motion directions conveyed by these responses. The simulations enabled us to make two predictions for the decoded representation.

First, we confirmed that if the population response reflects both spatial orientation and velocity cues, and the velocity response is relatively noisy, the decoded probability distribution is bimodal with two unequal peaks corresponding to the true and opposite direction (Supplementary Figure 7a). In contrast, a unimodal distribution is observed when decoding from a population that only represents velocity cues, and a bimodal distribution with two equally high peaks is obtained when decoding from a population response that reflects spatial orientation cues alone. As for the Bayesian observer model described in the main text, the shape of the decoded posterior depends (on average) on the relative amount of noise in the two signals (Supplementary Figure 20).

Second, the bimodal shape of the mean distribution across trials (decoded from responses that reflect both cues) is not an artefact of the aggregation. The locations of the peaks in the decoded posterior can be quantified on a trial-by-trial basis using a mixture of two von Mises basis functions. Supplementary Figure 7b shows the joint distribution of the locations of the larger and smaller peak in this mixture. Using this quantification, three clusters of trials are observed, with two components either co-located at the true motion direction (corresponding to a unimodal average posterior) or one of them located at the true and another located at the opposite motion direction (corresponding to a bimodal posterior). This is in sharp contrast to what is observed when decoding from a population response that represent only velocity cues (only one cluster of trials is observed, corresponding to a unimodal average posterior) or only orientation cues (two cluster of trials with one of the two components located at the true motion direction and another at the opposite one).

Simulated BOLD responses

To obtain predictions for the shape of the decoded posterior distribution, we simulated the responses from a population of velocity- and orientation-tuned neurons. We combined the responses in idealized voxels, the response of which is a simple sum of the signals from the underlying neuronal populations, combined with additional noise due to the fMRI measurements. Specifically, we assumed that the response of each of *N* voxels reflects a mixture of signals from two independent neural populations, a bimodally-tuned one and a unimodally-tuned one (corresponding to orientation-tuned and velocity-tuned neural populations, respectively), and noise. Accordingly, the BOLD response vector of size *N*, $\mathbf{b}_{tot}$, was modeled as the sum of three components:

$$\mathbf{b}_{tot} = \mathbf{b}_V + \mathbf{b}_O + \mathbf{b}_\epsilon \tag{1}$$

where $\mathbf{b}_V$ and $\mathbf{b}_O$ refer to the vectors of velocity-tuned and orientation-tuned components, respectively and $\mathbf{b}_\epsilon$ refers to the noise in BOLD responses due to other factors, such as non-neural fMRI-specific noise or neural responses uncorrelated with the orientation or velocity components. The velocity-tuned and orientation-tuned components are normally distributed with stimulus-dependent mean $\boldsymbol{\mu}_V(s)$ and $\boldsymbol{\mu}_O(s)$, and covariance $\boldsymbol{\Sigma}_V$ and $\boldsymbol{\Sigma}_O$, respectively:

$$\mathbf{b}_V \sim \mathcal{N}(\boldsymbol{\mu}_V(s), \boldsymbol{\Sigma}_V) \tag{2}$$

$$\mathbf{b}_O \sim \mathcal{N}(\boldsymbol{\mu}_O(s), \boldsymbol{\Sigma}_O) \tag{3}$$

The noise component $\mathbf{b}_\epsilon$ is normally-distributed with zero mean and covariance $\mathbf{\Sigma}_\epsilon$:

$$\mathbf{b}_\epsilon \sim \mathcal{N}(0, \mathbf{\Sigma}_\epsilon) \tag{4}$$

where $\mathbf{\Sigma}_\epsilon$ is a diagonal covariance matrix determining the noise specific to individual voxels with variance τ_i^2:

$$\mathbf{\Sigma}_\epsilon = \mathrm{diag}(\tau_1^2, \ldots, \tau_\mathrm{N}^2) \tag{5}$$

The mean response $\boldsymbol{\mu}_V(s)$ and $\boldsymbol{\mu}_O(s)$ of the velocity-tuned and orientation-tuned components is given by:

$$\boldsymbol{\mu}_V(s) = \sum_{k=1}^{K} \mathbf{W}_{V_k} g_k(s) \tag{6}$$

$$\boldsymbol{\mu}_O(s) = \sum_{k=1}^{K} \mathbf{W}_{O_k} g_k(s) \tag{7}$$

Here, $\mathbf{W}_V$ and $\mathbf{W}_O$ are weights, *K*=8, and *g* is a basis function:

$$g_k(s) = \max(0, \cos(s - \varphi_k))^5 \tag{8}$$

where φ_k is the preferred motion direction of basis function *k*. The mean response (as a function of *s*) can be thought of as the voxel's tuning function for the velocity and spatial orientation signals. The weights $\mathbf{W}_V$ are described by an *N* by *K* matrix of 1 by *K* unit vectors $\mathbf{w}_{V_{i*}}$ with *K* uniformly-distributed values so that for the *i*-th voxel:

$$\mathbf{w}_{V_{i*}} = z(\mathbf{v})^\mathrm{T} \tag{9}$$

$$\mathbf{v} \sim \mathcal{U}_K(0,1) \tag{10}$$

where $z(\mathbf{v})$ is the function that normalizes a vector by its Euclidean norm, so that for any vector $\mathbf{v}$:

$$z(\mathbf{v}) = \frac{\mathbf{v}}{\|\mathbf{v}\|_2} \tag{11}$$

For the spatial orientation-tuned component, the weights $\mathbf{W}_O$ are distributed in the same way as for the velocity-tuned component for half of the basis functions (indicated by vector **a**) and repeated for the other half, after which they are normalized to unit length:

$$\mathbf{w}_{O_{i*}} = z\left(\langle a_1 \ldots a_{K/2}, a_1 \ldots a_{K/2} \rangle^\mathrm{T}\right)^\mathrm{T} \tag{12}$$

$$\mathbf{a} \sim \mathcal{U}_{K/2}(0,1) \tag{13}$$

The covariance matrices $\mathbf{\Sigma}_V$ and $\mathbf{\Sigma}_O$ define the degree of (shared) noise in the voxels. Their structure depends on the degree of overlap in stimulus preference (indicated by $\mathbf{W}_V\mathbf{W}_V^\mathrm{T}$ and $\mathbf{W}_O\mathbf{W}_O^\mathrm{T}$) of the respective components, scaled by σ_V^2 and σ_O^2:

$$\mathbf{\Sigma}_V = \sigma_V^2 \mathbf{W}_V \mathbf{W}_V^T \tag{14}$$

$$\mathbf{\Sigma}_O = \sigma_O^2 \mathbf{W}_O \mathbf{W}_O^T \tag{15}$$

See van Bergen et al.[6] and van Bergen and Jehee[7] for additional derivations of, and rationale for, this particular covariance structure.

Following Supplementary Eq. 1-4, the total voxel response $\mathbf{b}_{tot}$ is a multivariate normally distributed variable:

$$\mathbf{b}_{tot} \sim N(\boldsymbol{\mu}_{tot}, \boldsymbol{\Sigma}_{tot}) \tag{16}$$

with mean response $\boldsymbol{\mu}_{tot}$ and covariance $\boldsymbol{\Sigma}_{tot}$ defined as:

$$\boldsymbol{\mu}_{tot} = \boldsymbol{\mu}_V + \boldsymbol{\mu}_O = \sum_{k=1}^{K} \left(\mathbf{W}_{V_k} + \mathbf{W}_{O_k}\right) g_k(s) \tag{17}$$

$$\boldsymbol{\Sigma}_{tot} = \boldsymbol{\Sigma}_V + \boldsymbol{\Sigma}_O + \boldsymbol{\Sigma}_\epsilon = \sigma_V^2 \mathbf{W}_V \mathbf{W}_V^{\mathrm{T}} + \sigma_O^2 \mathbf{W}_O \mathbf{W}_O^{\mathrm{T}} + \mathrm{diag}(\tau_1^2, \ldots, \tau_{\mathrm{N}}^2) \tag{18}$$

We simulated voxel responses for different levels of velocity and orientation noise with σ_V and σ_O varying from 0.1 to 1.3 in 0.2 steps. In each simulation, we generated the BOLD response for 500 voxels and 100 trials with randomly chosen stimuli for 10 observers. Voxel tuning preferences (determined by $\mathbf{W}_O$ and $\mathbf{W}_V$) were randomly and independently drawn for each voxel according to Eqs. S6-S13. The noise variance parameter τ_i^2 was normally distributed with mean μ_τ and variance $\sigma_\tau^2 = 0.035$ across voxels. We used different levels of μ_τ varying from 0.3 to 1.1 in 0.2 steps. To obtain posterior distributions, we inverted the generative model of voxel responses (Eqs. S1-S4, and a uniform prior) using Bayes rule. We obtained posterior distributions given the combined responses ($p(s|\mathbf{b}_{tot})$), velocity responses alone ($p(s|\mathbf{b}_V)$), and spatial orientation response alone ($p(s|\mathbf{b}_O)$). The results are presented in Supplementary Figure 7 and Supplementary Figure 20.

2. Deriving the posterior distribution for the Bayesian observer model.

Here, we provide the derivation for the likelihood function of the Bayesian observer (Eq. 21 in the main text) reproduced here for convenience:

$$L(s) = f_{VM}(s; x_V, \kappa_V)\big(f_{VM}(s; x_O, \kappa_O) + f_{VM}(s; x_O + \pi, \kappa_O)\big)$$

where s refers to the motion direction of the stimulus, x_V and x_O are the velocity and orientation measurements, and κ_V and κ_O are the associated precision parameters. This equation shows the sum of two products, each of which involves two von Mises distributions. In other words, it is a mixture distribution. Recall that a product of two VM distributions, 1 and 2, is an unnormalized von Mises distribution [8,9]:

$$f_{VM}(x; \theta_1, \kappa_1) f_{VM}(x; \theta_2, \kappa_2) = \frac{\exp(\kappa \cos(x - \theta))}{4\pi^2 I_0(\kappa_1) I_0(\kappa_2)} \tag{19}$$

where θ_1 and θ_2 are the peak locations of the two distributions and κ_1 and κ_2 refer to their precision. The location θ_{prod} of the resulting distribution is between the locations of the original distributions θ_1 and θ_2 with the relative position dependent on the ratio of two precisions ($\frac{\kappa_1}{\kappa_2}$):

$$\theta_{prod} = \theta_1 - \operatorname{atan2}\left(\sin(\theta_1 - \theta_2), \frac{\kappa_1}{\kappa_2} + \cos(\theta_1 - \theta_2)\right) \tag{20}$$

The precision of a product of two VM distributions (κ_{prod}) depends on their individual precisions (κ_1 and κ_2) and the distance between their locations ($\theta_1 - \theta_2$):

$$\kappa_{prod} = \sqrt{\kappa_1^2 + \kappa_2^2 + 2\kappa_1\kappa_2 \cos(\theta_1 - \theta_2)} \tag{21}$$

For the likelihood function described here, there are two such products in a mixture distribution. Thus, applying Supplementary Eq. 19, the likelihood (Eq. 21 in the main text) is described as a mixture of two von Mises distributions with locations θ_A and θ_B and precisions κ_A and κ_B computed per Supplementary Eq. 20 and 21:

$$L(s) = L_A(s) + L_B(s) \tag{22}$$

The first part (A) of the mixture is a product of the velocity likelihood and the first peak of the orientation likelihood:

$$L_A(s) = f_{VM}(s; x_V, \kappa_V) f_{VM}(s; x_O, \kappa_O) = \frac{\exp(\kappa_A \cos(s - \theta_A))}{4\pi^2 I_0(\kappa_O) I_0(\kappa_V)} \tag{23}$$

with mean and location given by:

$$\theta_A = x_V - \operatorname{atan2}\left(\sin(x_V - x_O), \frac{\kappa_V}{\kappa_O} + \cos(x_V - x_O)\right) \tag{24}$$

$$\kappa_A = \sqrt{\kappa_V^2 + \kappa_O^2 + 2\kappa_V\kappa_O \cos(x_V - x_O)} \tag{25}$$

The second part (B) of the mixture is a product of the velocity likelihood and the second peak from the orientation likelihood:

$$L_B(s) = f_{VM}(s; x_V, \kappa_V) f_{VM}(s; x_O + \pi, \kappa_O) = \frac{\exp(\kappa_B \cos(s - \theta_B))}{4\pi^2 I_0(\kappa_O) I_0(\kappa_V)} \quad (26)$$

Using trigonometric identities, it can be demonstrated that:

$$\theta_B = x_V + \text{atan2}\left(\sin(x_V - x_O), \frac{\kappa_V}{\kappa_O} - \cos(x_V - x_O)\right) \quad (27)$$

$$\kappa_B = \sqrt{\kappa_V^2 + \kappa_O^2 - 2\kappa_V \kappa_O \cos(x_V - x_O)} \quad (28)$$

Given that the denominator in Eqs. 23 and 26 does not depend on the stimulus, the posterior distribution is proportional to a sum of two exponents:

$$p(s|x_O, x_V) \propto \exp(\kappa_A \cos(s - \theta_A)) + \exp(\kappa_B \cos(s - \theta_B)) \quad (29)$$

After including the normalization constant, it can be shown that:

$$p(s|x_O, x_V) = w_A f_{VM}(s; \theta_A, \kappa_A) + w_B f_{VM}(s; \theta_B, \kappa_B) \quad (30)$$

where

$$w_A = \frac{I_0(\kappa_A)}{I_0(\kappa_A) + I_0(\kappa_B)} \quad (S31)$$

$$w_B = \frac{I_0(\kappa_B)}{I_0(\kappa_A) + I_0(\kappa_B)} \quad (S32)$$

Supplementary Equation 30 summarizes the posterior as a mixture of two von Mises distributions with weights defined by their relative precision. Each of the two peaks is a product of the velocity and orientation likelihoods. The precision and the location of the two peaks in the mixture depend on the precision and the location of the velocity and orientation components.

3. Additional information on the simulations provided in Supplementary Figures

Additional information for Supplementary Fig. 4

To quantify the predicted relationship between the decoded posterior distribution and the observer's behavioral responses for low coherence stimuli, we first fitted the Bayesian model to the behavioral estimates of the human observers in the follow-up study (low coherence stimuli); this gave us an estimate of the noise parameters of the underlying orientation and velocity likelihoods. We additionally estimated the amount of fMRI noise in the main experiment by fitting the model's parameters to the decoded posteriors and the observers' behavioral estimates in this experiment. We then used these values in our simulations, following the same procedures as discussed in the text (see Methods, 'Bayesian observer models'). We simulated both the decoded posterior distribution and the observer's behavioral response on 180000 trials (10000 trials for each of 18 observers). The simulated trials were subsequently divided over two bins: one for which the largest peak of the decoded posterior fell 'opposite' to the presented direction of motion (i.e., 170-190 degrees away from the presented direction of motion), and another bin for all the other trials. Similar results are obtained when selecting a larger orientation range for the 'opposite' bin. Within each bin, we then calculated the fraction of trials on which the model observer gave the opposite behavioral response. We did this for both the velocity-only model and the Bayesian model. As can be seen in the figure, both models predict an increase in opposite behavioral responses when coherence levels are low and uncertainty is high.

To see why also the velocity-only model predicts an increase in opposite behavioral responses for low coherence stimuli, consider that the velocity measurement fluctuates across trials because of random noise. Because the measurement fluctuates, so does the location of the likelihood's peak, and consequently, the Maximum Likelihood Estimate (MLE, i.e., the velocity-only readout). The trial-by-trial variance in the MLE is directly related to the variance in the observer's measurements and the width (variance) of the likelihood (see e.g., Eq. 11, 28). Thus, when uncertainty is high and the likelihood is sufficiently broad, the peak of the distribution (and associated behavioral response) will sometimes fall on the opposite direction of motion (even though the mean of the behavioral response distribution, which is computed across trials, is 0). The posterior distribution that is decoded from the velocity and orientation signals is strongly affected by the location of the velocity measurement (Eq. 21, 30). Consequently, when the velocity measurement falls opposite to the true direction of motion, the largest peak of the decoded posterior will likely also be located close to the opposite direction of motion, resulting in a link between the two.

Additional information for Supplementary Fig. 10

The simulations in this figure follow the same steps as the simulations in Fig. 2c, Supplementary Supplementary Figure 1, and Supplementary Figure 11. The observer's measurements are drawn independently from two von Mises distributions (one for velocity and one for orientation). We varied the variance parameters in our simulations to account for trial-by-trial fluctuations in uncertainty. For consistency with the simulations in Fig. 2c, on each trial two precision parameters κ_V and κ_O were first drawn independently from a log-normal distribution with $\mu_{neur} = 3.8$ and $\sigma_{neur} = 0.6$. These precision parameters were then converted to the variance parameters of a bivariate normal distribution, σ_V^2 and σ_O^2. Based on these variances, and for each trial, two measurements x_V and x_O were randomly drawn (wrapped in 360˚ and 180˚ circular spaces, respectively) from a bivariate wrapped normal circular distribution. For the `MAP readout` model, the likelihood was obtained numerically by computing the probability of observing measurements x_V and x_O for each stimulus s on a 720-step (0.5˚) grid. The posterior was calculated using Equation 19, and we selected the stimulus with the maximum a posteriori probability (MAP) as the behavioral response. For the `velocity-only readout ` model, the velocity measurement x_V was taken as the behavioral response, because this matches the maximum likelihood solution. The peak locations of the decoded posterior distribution were obtained as follows. We assumed that the fMRI measurements added noise to the observer's two measurements; the two fMRI noise values for a given trial were drawn from a bivariate wrapped normal

distribution centered on 0 and with correlation coefficient r_{noise}. We varied the amount of correlation between the two noise variables in our simulations, $r_{noise} = \{0.0, 0.3, 0.5, 0.7, 1.0\}$. The variance parameters σ'^2_V and σ'^2_O of the bivariate normal were obtained by converting two precision parameters, κ'_V, κ'_O, which were themselves drawn randomly from a log-normal distribution with $\mu_{MRI,velocity} = 0.9$ and $\sigma_{MRI,velocity} = 1.1$ for velocity, and $\mu_{MRI,orientation} = 1.4$ and $\sigma_{MRI,orientation} = 0.7$ for orientation. These latter parameter values are the same as for the simulations reported in the main text. The posterior distribution given voxel activity was subsequently obtained by inverting this generative model, where the likelihood was computed numerically (similar to above). The peak locations were determined in the same way as for Supplementary Figure 1.

Additional information for Supplementary Fig. 11

The simulations in this figure follow the same steps as the simulations in Fig. 2c, Supplementary Figure 1, and Supplementary Figure 10. The observer's measurements are drawn independently from two von Mises distributions (one for velocity and one for orientation). The precision parameters of the von Mises distributions varied across trials (because the amount of uncertainty fluctuates across trials), but unlike for the simulations reported in the main text, these precision parameters covaried. Specifically, for each trial, the precision parameters κ_V and κ_O were first drawn from a bivariate log-normal distribution with $\mu_{neur} = 3.8$ and $\sigma_{neur} = 0.6$ and with correlation coefficient $r_{\kappa_{neur}}$. We varied the strength of the correlation between the uncertainty parameters in our simulations, $r_{\kappa_{neur}} = \{0.0, 0.3, 0.5, 0.7, 1.0\}$. The precision parameters were then converted to the variance parameters of a bivariate normal distribution, σ^2_V and σ^2_O. Using these variances, for each trial, two measurements x_V and x_O (wrapped in 360˚ and 180˚ circular spaces, respectively) were drawn from the bivariate normal distribution. The rest of the simulations were done in the same way as for Supplementary Figure 1. In short, for the `MAP readout` model, the behavioral response was determined as the most likely stimulus given the measurements x_V and x_O, where the likelihood was calculated numerically on a 720-step (0.5˚) grid. For the `velocity-only readout` model, the behavioral response is identical to the velocity measurement x_V, as this is the maximum likelihood solution. The posterior distribution decoded from fMRI signals was obtained in the same was as for Supplementary Figure 18, and the peak locations were obtained following the procedures of Supplementary Figure 18, as well.

Additional information for Supplementary Fig. 18

The simulations in this figure follow the same steps as the simulations in Fig. 2c. However, instead of assuming that the observer's measurements are independently drawn from two von Mises distributions (one for velocity and one for orientation), a bivariate wrapped normal distribution was used with varying amounts of correlation, $r_{neur} = \{0.0, 0.3, 0.5, 0.7, 1.0\}$, between the measurements. The variance parameters of the bivariate normal distribution varied across trials in our simulations to account for trial-by-trial fluctuations in uncertainty. For consistency with the simulations in Fig. 2c, on each trial two precision parameters κ_V and κ_O were first drawn independently from a log-normal distribution with $\mu_{neur} = 3.8$ and $\sigma_{neur} = 0.6$. These precision parameters were then converted to the variance parameters of the bivariate normal distribution, σ^2_V and σ^2_O. Based on these variances and r, and for each trial, two measurements x_V and x_O (wrapped in 360˚ and 180˚ circular spaces, respectively) were then drawn from the bivariate normal distribution. For the `MAP readout` model, the likelihood was obtained numerically by computing the joint probability of observing measurements x_V and x_O for each stimulus s on a 720-step (0.5˚) grid. The posterior was obtained using Bayes rule and a flat prior (Eq. 19), and we selected the stimulus with the maximum a posteriori probability (MAP) as the behavioral response. For the `velocity-only readout` model, we took the velocity measurement x_V as the behavioral response, because this corresponds to the maximum likelihood solution. We assumed that the fMRI measurements added noise to the two measurements of the observer. That is, the two fMRI noise values x'_V and x'_O for a given trial were independently drawn from a bivariate wrapped normal distribution centered on 0. The variance parameters σ'^2_V and σ'^2_O of the bivariate normal were obtained by converting two precision parameters, κ'_V, κ'_O, which were themselves drawn randomly from a log-normal distribution with

$\mu_{MRI,velocity} = 0.9$ and $\sigma_{MRI,velocity} = 1.1$ for velocity, and $\mu_{MRI,orientation} = 1.4$ and $\sigma_{MRI,orientation} = 0.7$ for orientation. These parameter values are the same as for the simulations reported in the main text. The fMRI likelihood was obtained numerically by computing the joint probability of observing the fMRI measurements $x_V + x_V'$ and $x_O + x_O'$ for each stimulus *s* on a 720-step (0.5˚) grid. The decoded posterior distribution was calculated using Bayes rule and a flat prior. The peak locations of the simulated decoded posterior were estimated in the same way as those that were decoded from fMRI data (see Methods, *Analyses of the shape of the decoded distribution* for details). That is, a two-component mixture model was fitted to estimate the peak locations. The correlation of each peak location with the behavioral response was then estimated for trials where one of the peaks was closer to the true (-90° to 90°) and the other was closer to the opposite (90° to 270°) direction of motion.

Additional information for Supplementary Fig. 13

The posterior distribution of motion direction given simulated responses of bimodally-tuned direction selective neural populations was obtained as follows. We simulated voxel responses for a large number of neural populations with varying levels of direction selectivity. The Direction Index (DI) of each population was defined as $DI = 1 - \frac{r_{np}}{r_p}$, where r_p is the mean response to the preferred direction, and r_{np} is the mean response to the opposite direction. We used a realistic distribution of Direction Indices (*DI*) across the populations that matched the empirically observed distribution in area V1 of alert monkeys [4]. Voxels were defined in the same way as the orientation-tuned voxels described in Supplementary Methods, section 1 (Supplementary Eq. 12) with weights $\mathbf{W}$, but now the second half of their weights was multiplied by the underlying population's *DI* to reflect their selectivity. *DI* values were drawn randomly from a discrete distribution with values {0.55, 0.65, 0.75, 0.85, 0.95} and probabilities {0.09, 0.10, 0.11, 0.16, 0.55}, which corresponds to the empirically observed distribution calculated across V1 layers (Figure 5 in [4]). We focused on direction selective populations only, as defined by [4] (i.e., *DI* > 0.5). The preferred direction of motion of the neural population was distributed uniformly across the direction space for each DI value. Using these parameters, the voxel responses were randomly drawn from a multivariate normal distribution with mean $\boldsymbol{\mu}_{tot} = \sum_{k=1}^{K} \mathbf{W}_k g_k(s)$ and covariance matrix $\boldsymbol{\Sigma} = \sigma^2 \mathbf{W}\mathbf{W}^T + \mathrm{diag}(\tau_1^2, \dots, \tau_N^2)$. The posterior distribution given the voxel response was computed by inverting the generative model and using a flat prior. Supplementary Fig. 20 shows the obtained results.

Supplementary References

1. Ress, D., Backus, B. T. & Heeger, D. J. Activity in primary visual cortex predicts performance in a visual detection task. *Nat. Neurosci.* **3**, 940–945 (2000).
2. Edwards, M. & Crane, M. F. Motion streaks improve motion detection. *Vision Res.* **47**, 828–833 (2007).
3. Laquitaine, S. & Gardner, J. L. A Switching Observer for Human Perceptual Estimation. *Neuron* **97**, 462-474.e6 (2018).
4. Gur, M., Kagan, I. & Snodderly, D. M. Orientation and direction selectivity of neurons in V1 of alert monkeys: Functional relationships and laminar distributions. *Cereb. Cortex* **15**, 1207–1221 (2005).
5. Tohmi, M., Tanabe, S. & Cang, J. Motion Streak Neurons in the Mouse Visual Cortex. *Cell Rep.* **34**, 108617 (2021).

6. van Bergen, R. S., Ma, W. J., Pratte, M. S. & Jehee, J. F. M. Sensory uncertainty decoded from visual cortex predicts behavior. *Nat. Neurosci.* **18**, 1728–1730 (2015).
7. van Bergen, R. S. & Jehee, J. F. M. Modeling correlated noise is necessary to decode uncertainty. *NeuroImage* **180**, 78–87 (2018).
8. Murray, R. F. & Morgenstern, Y. Cue combination on the circle and the sphere. *J. Vis.* **10**, 15–15 (2010).
9. Kalm, K. & Norris, D. Visual recency bias is explained by a mixture model of internal representations. *J. Vis.* **18**, 1 (2018).
