# Supplementary material for: Motion direction is represented as a bimodal probability distribution in the human visual cortex
Source: Nat Commun. 2023 Nov 22;14:7634. doi: 10.1038/s41467-023-43251-w (PMC10665457; doi:10.1038/s41467-023-43251-w)
Supplement: Supplementary file 3 — Reporting Summary [file 41467_2023_43251_MOESM3_ESM.pdf]

nature portfolio

Corresponding author(s): Andrey Chetverikov, Janneke Jehee

Last updated by author(s): Oct 3, 2023

Reporting Summary

Nature Portfolio wishes to improve the reproducibility of the work that we publish. This form provides structure for consistency and transparency in reporting. For further information on Nature Portfolio policies, see our Editorial Policies and the Editorial Policy Checklist.

Statistics

For all statistical analyses, confirm that the following items are present in the figure legend, table legend, main text, or Methods section.

n/a	Confirmed	
☐	☒	The exact sample size (*n*) for each experimental group/condition, given as a discrete number and unit of measurement
☐	☒	A statement on whether measurements were taken from distinct samples or whether the same sample was measured repeatedly
☐	☒	The statistical test(s) used AND whether they are one- or two-sided<br>*Only common tests should be described solely by name; describe more complex techniques in the Methods section.*
☐	☒	A description of all covariates tested
☐	☒	A description of any assumptions or corrections, such as tests of normality and adjustment for multiple comparisons
☐	☒	A full description of the statistical parameters including central tendency (e.g. means) or other basic estimates (e.g. regression coefficient) AND variation (e.g. standard deviation) or associated estimates of uncertainty (e.g. confidence intervals)
☐	☒	For null hypothesis testing, the test statistic (e.g. *F*, *t*, *r*) with confidence intervals, effect sizes, degrees of freedom and *P* value noted<br>*Give P values as exact values whenever suitable.*
☐	☒	For Bayesian analysis, information on the choice of priors and Markov chain Monte Carlo settings
☐	☒	For hierarchical and complex designs, identification of the appropriate level for tests and full reporting of outcomes
☐	☒	Estimates of effect sizes (e.g. Cohen's *d*, Pearson's *r*), indicating how they were calculated

Our web collection on statistics for biologists contains articles on many of the points above.

Software and code

Policy information about availability of computer code

Data collection	Matlab 2017b
Data analysis	Matlab 2017b, FSL 5.0.9, freesurfer 5.3, R 3.5.3 (packages used are listed in the source code), Python 2.7 (packages used are listed in the source code), custom code TAFKAP decoding algorithm: https://github.com/jeheelab/TAFKAP; see van Bergen et al., 2015, Nature Neuroscience; van Bergen & Jehee, 2018, NeuroImage; van Bergen & Jehee, 2021, bioRxiv)

For manuscripts utilizing custom algorithms or software that are central to the research but not yet described in published literature, software must be made available to editors and reviewers. We strongly encourage code deposition in a community repository (e.g. GitHub). See the Nature Portfolio guidelines for submitting code & software for further information.

Data

Policy information about availability of data

All manuscripts must include a data availability statement. This statement should provide the following information, where applicable:

- Accession codes, unique identifiers, or web links for publicly available datasets
- A description of any restrictions on data availability
- For clinical datasets or third party data, please ensure that the statement adheres to our policy

Preprocessed behavioral and fMRI data of individual participants generated in this study have been deposited in the Donders Repository database: http://dx.doi.org/10.34973/yk4k-tp41. This includes the data necessary to reproduce the figures. These data are available open access. The raw fMRI data are protected

and are available upon request from the last author (Janneke F.M. Jehee) due to data privacy regulations. Requests for data will be answered within a reasonable timeframe (1 month).

Research involving human participants, their data, or biological material

Policy information about studies with human participants or human data. See also policy information about sex, gender (identity/presentation), and sexual orientation and race, ethnicity and racism.

Reporting on sex and gender	Sex and gender were not considered in the study design, as it is unlikely that these factors will underlie differences in visual cortical processing. The aggregated information about participants' sex is provided in the text (10 female, 8 male participants).
Reporting on race, ethnicity, or other socially relevant groupings	Race, ethnicity, or other socially relevant groupings were not considered in the study design, as it is unlikely that these factors will underlie differences in visual cortical processing. The data about these characteristics were not collected.
Population characteristics	18 participants (aged 18-32, ten female) with normal or corrected to normal vision participated in the study.
Recruitment	Participants were recruited through the university recruitment system (SONA). It is unlikely that a self-selection bias or other biases will underlie differences in visual cortical processing.
Ethics oversight	This study complies with all relevant ethical regulations and was approved by the local ethics committee (Commissie Mensgebonden Onderzoek Regio Arnhem-Nijmegen, The Netherlands; Protocol CMO2014/288). Participants provided written informed consent before participation.

Note that full information on the approval of the study protocol must also be provided in the manuscript.

Field-specific reporting

Please select the one below that is the best fit for your research. If you are not sure, read the appropriate sections before making your selection.

☒ Life sciences ☐ Behavioural & social sciences ☐ Ecological, evolutionary & environmental sciences

For a reference copy of the document with all sections, see nature.com/documents/nr-reporting-summary-flat.pdf

Life sciences study design

All studies must disclose on these points even when the disclosure is negative.

Sample size	18 participants were recruited in a repeated-measurements design with an average of 792 trials per participant in the main study. This should have provided above 99% sensitivity based on the previously reported decoding performance (van Bergen et al., 2015, Nature Neuroscience).
Data exclusions	Trials on which the participant's behavioral error was more than three standard errors away from the bias-corrected mean of that participant were marked as guesses and excluded from further analysis (0.66%).
Replication	We implemented an ideal observer model (i.e., normative computational model). This resulted in a number of quantitative predictions, each of which we experimentally tested in several independent ways. All of our predictions were supported by the empirical data.
Randomization	We used a within-subjects design, so no allocation into experimental groups was necessary.
Blinding	We used a within-subjects design so blinding was not necessary.

Reporting for specific materials, systems and methods

We require information from authors about some types of materials, experimental systems and methods used in many studies. Here, indicate whether each material, system or method listed is relevant to your study. If you are not sure if a list item applies to your research, read the appropriate section before selecting a response.

Materials & experimental systems

n/a	Involved in the study
☒	☐ Antibodies
☒	☐ Eukaryotic cell lines
☒	☐ Palaeontology and archaeology
☒	☐ Animals and other organisms
☒	☐ Clinical data
☒	☐ Dual use research of concern
☒	☐ Plants

Methods

n/a	Involved in the study
☒	☐ ChIP-seq
☒	☐ Flow cytometry
☐	☒ MRI-based neuroimaging

Magnetic resonance imaging

Experimental design

Design type

Task; slow event-related design

Design specifications

Three main fMRI sessions in per participant. 39-49 runs in total per participant. 18 trials per run (16.5 s/trial + 12 s fixation at the start of the run and 12 s at the end). Additionally, each scan session included two functional localizer runs, in which a localizer stimulus consisted of moving dots presented within a circular aperture (described by the same parameters as the main experiment) was presented in seven 12-s intervals ('stimulus interval'), interleaved with fixation intervals of equal duration.

Behavioral performance measures

We recorded the position of the response bar, as well as the timing of the first and last button presses, in each response window. Participants generally performed well on the task, with a mean absolute behavioral estimation error of M = 6.18°, 95% CI = [5.72°, 6.62°]. In general, participants finished adjusting their orientation and confidence responses well before the end of the response windows (4.5 s each), taking on average 2.9 s to respond (SD = 0.30 s).

Acquisition

Imaging type(s)

Functional, structural

Field strength

3T

Sequence & imaging parameters

For anatomical reference, a high-resolution T1-weighted image was collected at the start of each session (3D MPRAGE, TR: 2300 ms, TI: 1100 ms, TE: 3 ms, flip angle: 8 degrees, FOV: 256 x 256 mm, 192 saggital slices, 1-mm isotropic voxels).

B0 field inhomogeneity maps (TR: 653 ms, TE: 4.92 ms, flip angle: 60 degrees, FOV: 256 x 256 mm, 68 transversal slices, 2-mm isotropic voxels, interleaved slice acquisition) were acquired.

Functional data were acquired using a multi-band accelerated gradient-echo EPI protocol, in 68 transversal slices covering the whole brain (TR: 1500 ms, TE: 38.60 ms, flip angle: 75 degrees, FOV: 210 x 210 mm, 2-mm isotropic voxels, multiband acceleration factor: 4, interleaved slice acquisition).

Area of acquisition

Whole brain

Diffusion MRI ☐ Used ☒ Not used

Preprocessing

Preprocessing software

FSL 5.0.9 and freesurfer 5.3 were used for preprocessing of MRI data in a pipeline created with nipype in Python. FSL's MCFLIRT was used for motion correction in three passes with 12 DOF. Slow drifts in the BOLD signal were removed using FSL's nonlinear high-pass temporal filter (fslmaths) with a cut-off period of 50 s. Freesurfer's longitudinal processing stream (recon-all) was used to coregister the volumes between different sessions. FSL's scripts (fsl_prepare_fieldmap and epi_reg), were applied to preprocess a fieldmap and unwarp EPI data, and to register the unwarped data to the within-session anatomical reference image.

Normalization

The data were analyzed within subjects, so no normalization was necessary.

Normalization template

The data were analyzed within subjects, so no normalization was necessary.

Noise and artifact removal

The following nuisance regressors were used: an intercept regressor per run, 24 motion regressors based on the transformation parameters obtained from the motion correction algorithm. Motion regressors included raw displacement parameters (3 rotations + 3 translations), the displacement parameters squared, their temporal derivatives, and gradients. For the multivariate and ROI-based univariate analyses, nuisance signals were removed from the BOLD signal prior to further analyses, and no further artifact removal was applied.

Volume censoring

No volume censoring was performed.

Statistical modeling & inference

Model type and settings

We performed multivariate analyses based on a previously published, generative-model based decoding technique (TAFKAP decoding algorithm: https://github.com/jeheelab/TAFKAP; see van Bergen et al., 2015, Nature Neuroscience; van Bergen & Jehee, 2018, NeuroImage; van Bergen & Jehee, 2021, bioRxiv). Using this technique, and for each individual subject, we extracted from BOLD activity a probability distribution over stimulus motion direction on a trial-by-trial basis. 'Decoded uncertainty' was quantified as the entropy of the decoded distribution.

Effect(s) tested

We tested the relationship between decoded uncertainty and behavioral variability (variance of response errors), both corrected and uncorrected for differences in presented stimulus motion direction using Bayesian hierarchical regression. We ran benchmark tests for our decoding approach, testing the relationship between decoded and presented stimulus motion direction (circular correlation), between decoded uncertainty and stimulus distance to cardinal (Bayesian hierarchical regression), and between decoded uncertainty and behavioral variability (Bayesian hierarchical regression). For the analyses quantifying the shape of the decoded posterior distribution, we used model-fitting approach with Jensen-Shannon divergence (JSD, a symmetrized version of the Kullback–Leibler divergence) as the fitting criterion. For the analysis of across-trial distribution of peak locations, we fitted two bivariate von Mises mixture models to this distribution using the BAMBI package in R.

Specify type of analysis: ☐ Whole brain ☒ ROI-based ☐ Both

Anatomical location(s)

Regions of interests (ROIs) were defined using standard retinotopic procedures (visual areas V1, V2, V3AB, and hV4) and a functional localizer (hMT+). Specifically, for each individual participant, hMT+ was delineated manually on the inflated cortical surface as the area that included voxels responding more strongly to both 1) moving (i.e., optic flow patterns) rather than static dots ($p < .05$, FDR-corrected), and 2) coherent rather than random motion ($p < .05$, FDR-corrected; see Supplementary Fig. 2 for an example participant; the details in Methods). Unless otherwise specified, individual ROIs were combined into a single ROI for the main analyses.

Statistic type for inference

(See Eklund et al. 2016)

No voxel-wise or cluster-wise analyses were applied.

Correction

Not applicable.

Models & analysis

n/a	Involved in the study
☒	☐ Functional and/or effective connectivity
☒	☐ Graph analysis
☐	☒ Multivariate modeling or predictive analysis

Multivariate modeling and predictive analysis

We made use of a previously published, generative-model based, probabilistic decoding technique (TAFKAP decoding algorithm: https://github.com/jeheelab/TAFKAP; see van Bergen et al., 2015, Nature Neuroscience; van Bergen & Jehee, 2018, NeuroImage; van Bergen & Jehee, 2021, bioRxiv). From samples of BOLD activity, we extracted a probability distribution over motion directions. Within the ROI (areas V1, V2, V3AB, hV4, and hMT+ combined), we selected all voxels that were activated by the functional localizer stimulus while surviving a lenient statistical threshold ($p < 0.01$, uncorrected). The time series of each selected voxel was subsequently z-normalized with respect to corresponding trial time points in the same run. Activation patterns for each trial were obtained by averaging over the first 4.5 s of each trial, after adding a 3 s temporal shift to account for hemodynamic delay. A leave-one-run-out cross-validation procedure was used for model training and testing. The independent variable (for training) was the presented stimulus orientation.

Additional benchmark analyses verified that 1) motion direction decoding performance was well above chance levels, 2) decoded uncertainty was lower for cardinal compared to oblique directions, 3) decoded uncertainty predicted behavioral variability, both within and across motion directions, 4) the results remain stable for different number of voxels included in the analyses and in individual ROIs.
